# Diagnostic Value and Interobserver Reproducibility of Histopathological Features in Fumarate Hydratase-Deficient Uterine Leiomyomas

**DOI:** 10.3390/diagnostics15233006

**Published:** 2025-11-26

**Authors:** Aleksandra Rogozhina, Alina Badlaeva, Anna Tregubova, Aleksandra Asaturova, Maria Kuznetsova, Gennady Sukhikh

**Affiliations:** 1 National Medical Research Center for Obstetrics, Gynecology and Perinatology Named After Academician V.I. Kulakov of the Ministry of Health of Russia, Bldg. 4, Oparina Street, 117513 Moscow, Russia; a_badlaeva@oparina4.ru (A.B.); a_tregubova@oparina4.ru (A.T.); a.asaturova@gmail.com (A.A.); m_kuznetsova@oparina4.ru (M.K.); gennadiy_sukhikh@inbox.eu (G.S.); 2 Department of Pathological Anatomy and Clinical Pathological Anatomy, Pirogov Russian National Research Medical University, Bldg. 1, Ostrovitianov Street, 117997 Moscow, Russia

**Keywords:** fumarate hydratase-deficient leiomyoma, eosinophilic globules, atypical leiomyoma, interobserver reproducibility

## Abstract

**Background/Objectives:** Fumarate hydratase-deficient leiomyomas (dFH-LMs) are a rare subtype of uterine smooth muscle tumors (USMTs) with implications for hereditary leiomatosis and renal cell carcinoma (HLRCC). Although several morphologic clues have been proposed, their diagnostic reproducibility is poorly defined. We aimed to determine the diagnostic significance of histopathologic features associated with fumarase deficiency and the reproducibility of key morphologic criteria for defining dFH-LMs. **Methods:** A retrospective analysis was performed on 45 USMTs that were initially classified as atypical leiomyomas (ALMs). The cohort comprised patients aged 21 to 75 years who had surgery at one tertiary medical care center from 2016 to 2025. Hematoxylin–eosin (H&E) slides were independently examined by three pathologists for features associated with FH deficiency, including eosinophilic globules, staghorn-like vessels, diffuse nuclear atypia, “bizarre” nuclei, and prominent nucleoli with halos. Molecular status was determined by immunohistochemistry (IHC) for fumarate hydratase (FH) and S-(2-succino)-cysteine (2SC). Interobserver agreement was quantified using Fleiss’s *κ* and Cohen’s *κ*. **Results**: Loss of FH expression was detected in 26/45 tumors (57.7%). Eosinophilic globules occurred in 88.5% of dFH-LMs, but only in 15.8% of ALMs (*p* < 0.001). By majority consensus (≥2 of 3 reviewers), the eosinophilic globules predicted FH deficiency with a sensitivity of 88.0% and a specificity of 85.0%; interobserver reproducibility was substantial (*κ* = 0.63). Staghorn-like vessels occurred in 73.1% of dFH-LMs vs. 26.3% of ALMs (*p* = 0.02) and diffuse nuclear atypia (84.6%) was also more frequent in dFH-LMs (*p* = 0.01). Patients with dFH-LMs were significantly younger than those with ALMs (Median, 34 vs. 41 years). **Conclusions:** Eosinophilic globules, staghorn-like vessels and diffuse nuclear atypia have been shown to have high diagnostic value and could be considered morphologic indicators of dFH-LMs. The substantial interobserver reproducibility of eosinophilic globules makes this feature particularly promising for routine clinical practice.

## 1. Introduction

Although uterine leiomyomas are the most common benign tumors of the female reproductive system, their fumarate hydratase-deficient variant (dFH-LM) remains one of the most diagnostically challenging subtypes for pathologists [1]. This subtype of leiomyoma can occur sporadically due to a somatic mutation in the fumarate hydratase (FH) gene, while FH mutations in the germline are associated with hereditary leiomyomatosis and renal cell carcinoma syndrome (HLRCC) [2]. Although they are rare and account for approximately 0.5–2% of uterine smooth muscle tumors (USMTs), their detection is of great importance for both differential diagnosis and genetic counseling [3,4]. This differential diagnosis is critical as it includes uterine sarcomas, for which advanced imaging such as Magnetic Resonance Imaging (MRI) plays a significant role in preoperative assessment, although histological examination remains the gold standard for definitive diagnosis [5].

From a morphologic point of view, dFH-LM often has distinctive, albeit sometimes subtle, features, e.g., staghorn-like vessels, nuclear atypia and eosinophilic globules [6,7,8,9].

However, these features are not always present simultaneously, and their overlap with atypical leiomyomas (ALMs) often leads to diagnostic uncertainty [2,10].

This diagnostic instability presents challenges. In patients with germline FH mutations, dFH-LM may be the first manifestation of HLRCC, a syndrome associated with aggressive renal cell carcinoma that requires early genetic counseling and surveillance [2,9,11,12,13].

Despite the recognition of these morphological markers, the reproducibility of their evaluation by other observers has not yet been sufficiently researched. In particular, although eosinophilic globules are widely cited as one of pathognomonic features of FH deficiency, their consistent identification by different pathologists has not been systematically studied. Some authors have suggested that these inclusions may be underestimated, especially in tumors with overlapping features, limiting their practical diagnostic utility [14,15]. Therefore, the aim of the present study was to determine the interobserver reproducibility of eosinophilic globules in dFH-LM and to evaluate their predictive value for diagnosis.

## 2. Materials and Methods

**Figure 1:**
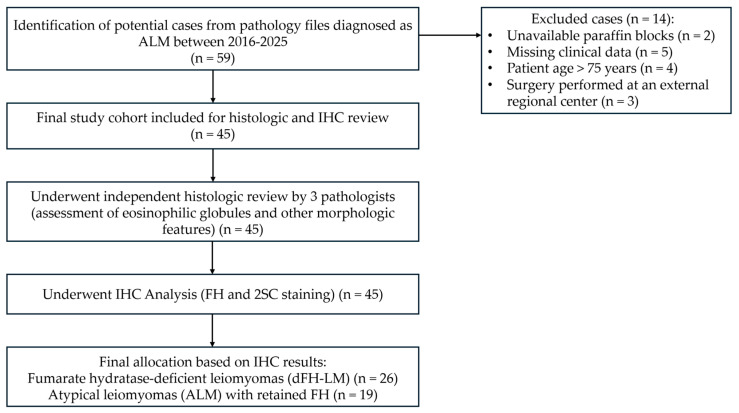
A study flowchart. ALM = Atypical leiomyoma, IHC = Immunohistochemistry, FH = Fumarate hydratase, 2SC = S-(2-succino) cysteine, dFH-LM = Fumarate hydratase-deficient leiomyoma.

A computerized search of the surgical pathology consultation and inpatient files at the Research Center for Obstetrics, Gynecology, and Perinatology (Moscow, Russia) was performed for all USMTs diagnosed as ALM between 2016 and 2025. All cases with original slides, paraffin blocks, and clinical follow-up data were included in the study. Cases were from patients aged 21–75 years who underwent surgery at a single tertiary medical center. All cases were retrieved from the histological archive of the 1st pathology department (Figure 1).

All hematoxylin–eosin (H&E) slides were examined by three pathologists (A.R., A.A., A.T.), paying attention to morphologic features previously associated with fumarase deficiency. The following variables were recorded: cellularity relative to the adjacent myometrium (categorized as hypercellular or normocellular), distribution of nuclear atypia (focal or diffuse), and the grade of nuclear atypia (scored as low (1+), intermediate (2+), or high (3+)). The following qualitative features were also assessed: presence or absence of eosinophilic globules, “bizarre” nuclei (defined as large, irregular nuclei visible with a 10× objective), presence or absence of nucleoli, and staghorn-like vessels (present or absent) [1,2,4,6,8]. In addition, the reviewers also recorded the presence of conspicuous “alveolar-type” edema and any tumor necrosis [1,4,8,15].

**Figure 2:**
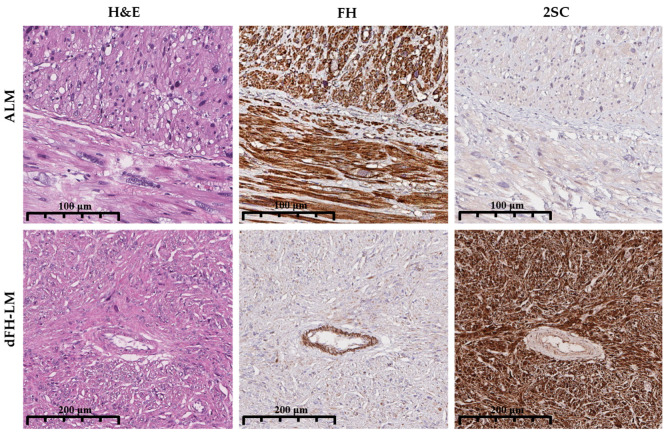
Comparative immunohistochemical features of ALM and dFH-LM. **Top row** (ALM): Hematoxylin and eosin (H&E) staining demonstrates an atypical smooth muscle proliferation. FH IHC shows retained cytoplasmic expression in tumor cells, with positive internal control staining in endothelial cells. 2SC IHC is negative in tumor cells, with only background staining evident. **Bottom row** (dFH-LM): H&E staining reveals a variably fascicular tumor with cytologic atypia. FH IHC demonstrates complete loss of expression in tumor cells, while internal control endothelial staining is preserved. 2SC IHC shows strong (3+), diffuse nuclear and cytoplasmic positivity in tumor cells (magnification 200× for all panels).

Clinical and gross parameters were abstracted, including patient age, tumor size, multiplicity (single or multiple nodules), and a history of recurrence. Clinical histories were reviewed to capture prior malignancy status. Immunohistochemistry (IHC) for FH (1:1000, clone EPR21104, Abcam, Cambridge, MA, USA) and S-(2-succino) cysteine (2SC) (1:100, polyclonal, Discovery, Discovery, Cambridge, UK) was performed on 4-µm sections from representative FFPE blocks using the VENTANA UltraView DAB IHC Detection Kit (Ventana Medical Systems, Oro Valley, AZ, USA) on a BenchMark XT automated stainer (Ventana Medical Systems, Oro Valley, AZ, USA). Three pathologists (A.R., A.A., A.T.) reviewed the IHC slides. FH staining was scored qualitatively as retained (positive) or lost (negative) relative to internal controls (endothelial/stromal cells). 2SC staining was evaluated for intensity (1+ to 3+) and distribution (nuclear–cytoplasmic vs. cytoplasmic-only), and only diffuse, 3+ nucleocytoplasmic staining was considered positive (Figure 2) [1,3,7,8].

**Figure 3:**
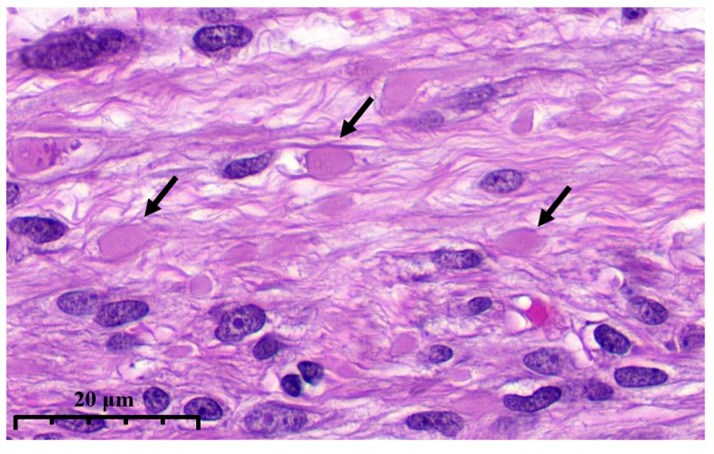
High magnification illustrates eosinophilic globules (arrows) in dFH-LM (previously unpublished, original photo) with H&E staining (magnification 400×).

To standardize the detection of eosinophilic globules—a hallmark of dFH-LM—histologic slides were independently evaluated by three pathologists. Because there are no recognized, widely accepted diagnostic criteria, we first assembled a study-specific training/familiarization set from slides published in publicly available peer-reviewed articles (https://dfh-lm.tilda.ws/, accessed on 1 September 2025). We adopted the following operational definition of eosinophilic globules: easily identifiable, round, homogeneous, dense/glassy eosinophilic intracellular or extracellular inclusions that may have a rhabdoid appearance at high magnification; typical size approximately equal to or slightly larger than the nucleus of a spindle-shaped smooth muscle cell; usually multiple; without surrounding inflammatory or fibrotic reaction (Figure 3). All histologic assessments were performed excluding IHC results.

Statistical analysis was performed in GraphPad Prism 9.3.1 (Dotmatics, Boston, MA, USA). Continuous and categorical variables were compared using the Mann–Whitney U test and Fisher’s exact test, respectively. Finally, an adjustment for multiplicity was performed for the resulting *p*-values of all univariate tests using the Holm-Bonferroni procedure. Interobserver agreement in the assessment of eosinophilic globules was quantified using Fleiss’s *κ* (overall) and pairwise Cohen’s *κ* statistics, interpreted as follows: *κ* = 0–0.40, none to minimal agreement; 0.41–0.60, moderate; 0.61–0.80, substantial; and 0.81–1.00, near perfect agreement. The reproducibility of the eosinophilic globules was assessed using a majority consensus method (≥2 of 3 reviewers) with simultaneous validation against the IHC results. To evaluate the diagnostic performance of eosinophilic globules for the prediction of dFH-LM, we calculated sensitivity, specificity, positive predictive value (PPV) and negative predictive value (NPV). The following formulas were used:

$$Specificity=\frac{true\;negative}{true\;negative+false\;positive}\;\times \;100$$

$$Sensitivity=\frac{true\;positive}{true\;positive+false\;negative}\times 100$$

$$PPV=\frac{true\;positive}{true\;positive+false\;positive}\times 100$$

$$NPV=\frac{true\;negative}{true\;negative+false\;negative}\times 100$$

Statistical significance was set at *p* < 0.05 (two-sided).

## 3. Results

According to the results of the IHC study, fumarase deficiency was identified in more than half of the cases of leiomyomas previously diagnosed as ALM (26 of 45 cases, 57.7%). The most diagnostically significant morphological features for the identification of dFH-LM were eosinophilic globules, staghorn-like vessels and the presence of diffuse nuclear atypia in the tumor.

**Figure 4:**
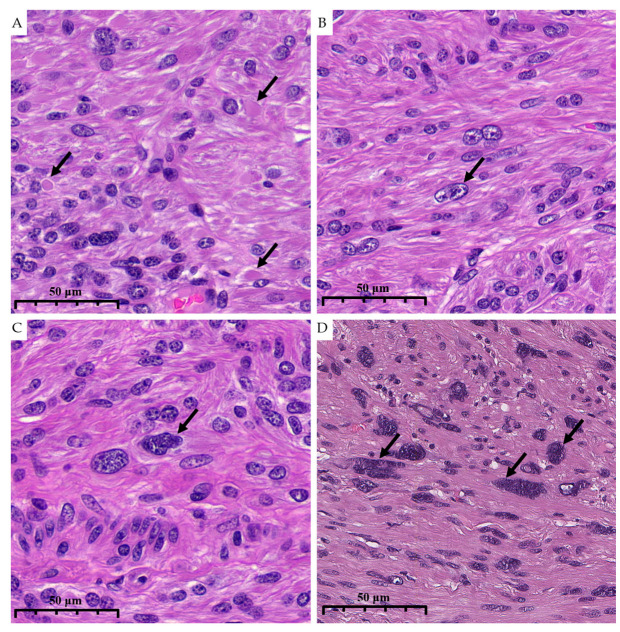
High magnification illustrates morphological characteristics of dFH-LM with H&E staining (previously unpublished, original photo). (**A**). Eosinophilic globules (arrow). (**B**). Nucleoli (arrow). (**C**,**D**). “Bizarre” nuclei (arrows) (magnification 400×).

Thus, eosinophilic globules were present in 88.5% of dFH-LMs and only in 15.8% of ALMs (*p* < 0.001). When defined by majority consensus (≥2 of 3 pathologists), eosinophilic globules predicted FH deficiency with a sensitivity of 88.0% and a specificity of 85.0%; PPV and NPV were 88.0% and 85.0%, respectively (Figure 4A). The interobserver reproducibility of eosinophilic globules was substantial (Fleiss’s *κ* = 0.63; 95% CI 0.44–0.81); the pairwise Cohen’s *κ* values are shown in Table 1.

The interobserver variance of the eosinophilic globules identified by the three pathologists and the FH/2SC staining are shown in Appendix A (Table A1).

**Figure 5:**
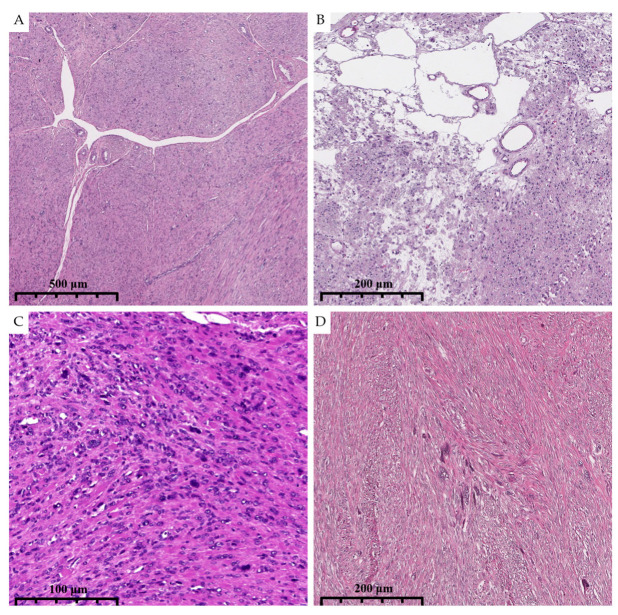
Photomicrographs show the histologic and cytological features of dFH-LM with H&E staining (previously unpublished, original photos). (**A**). Prominent staghorn-like vessels are present. (**B**). “Alveolar-type” edema. (**C**,**D**). Diffuse and focal distribution of nuclear atypia. (magnification 50× and 100×).

Staghorn-like vessels were observed in 73.1% of dFH-LMs versus 26.3% of ALMs (*p* = 0.02), yielding a sensitivity of 88.0% and a specificity of 83.3% for predicting FH deficiency (Figure 5A).

The distribution of nuclear atypia further distinguished the groups—dFH-LMs had the most diffuse atypia (84.6%), while ALMs were more likely to have focal atypia (63.2%) (*p* = 0.01); sensitivity and specificity for this criterion were 84.0% and 62.5%, respectively (Figure 5C,D).

In addition, dFH-LMs were statistically significantly more common in younger patients. The median age in the dFH-LM group was 34 years (interquartile range 27–39) versus 41 years (interquartile range 38–44) in the ALM group (*p* = 0.03).

Additional trends included a more frequent marked nuclear atypia in dFH-LM and a higher prevalence of conspicuous nucleoli (63% vs. 37%) (Figure 4B). Upon reviewing the health records of this group, we found no evidence of any prior cancer diagnoses. No other clinicopathologic variables differed significantly between the two groups. General clinical and histologic information for dFH-LM and ALM is presented in Table 2 and Figure 4 and Figure 5.

## 4. Discussion

USMTs are mesenchymal neoplasms that arise from smooth muscle cells. Although it is widely accepted that these tumors represent a heterogeneous group, the exact pathogenic mechanisms driving their development are still not fully understood. As described in the literature, USMTs exhibit considerable tissue heterogeneity and comprise several histological subtypes [1,16,17,18]. dFH-LM is a rare but clinically important subtype of smooth muscle mesenchymal neoplasm that arises after inactivation of the FH gene [1,4,19].

FH deficiency is a rare metabolic disorder that disrupts the tricarboxylic acid cycle (citric acid/Krebs), a central pathway of cellular energy production [20]. Biochemically, FH catalyzes the reversible hydration of fumarate to malate. Pathogenic variants of the FH gene abolish or diminish this catalytic activity, leading to intracytoplasmic accumulation of fumarate and widespread metabolic dysfunction that impairs mitochondrial respiration and ATP synthesis. It is important to note that such metabolic dysfunction is a biologically plausible basis for tumor development in a subset of smooth muscle neoplasms [11,12,13,19,21].

Within the citric acid cycle, acetyl-CoA is oxidized to CO_2_ and reducing equivalents (NADH, FADH_2_) drive the electron transport chain to generate ATP. Disruption of this cycle in FH deficiency reduces oxidative ATP production and forces a compensatory reliance on glycolysis, a less efficient pathway that increases lactate production—an adaptive shift often referred to as the Warburg effect and frequently documented in cancer biology [21,22,23,24]. Consistent with this mechanistic framework, dFH-LMs can be considered tumors that exploit a metabolic switch for growth advantage.

FH mutations have been detected in numerous tumor entities, most notably in HLRCC, a syndrome defined by a spectrum of benign and malignant neoplasms [1,15]. Excess fumarate in FH-deficient cells inhibits prolyl hydroxylases, thereby stabilizing hypoxia-inducible factor and activating transcriptional programs that promote angiogenesis, proliferation and survival under hypoxic stress. This represents a direct mechanistic link between the loss of FH and oncogenic signaling. In addition to its metabolic role, fumarase also appears to exert tumor suppressive functions. Specifically, accumulated fumarate acts as an oncometabolite that inhibits histone and DNA demethylases, causing epigenetic reprogramming and widespread transcriptional changes that promote tumor development and progression [4,22]. Overall, deficient FH-driven tumorigenesis can be considered to reflect the convergence of metabolic, hypoxic and epigenetic pathways.

From a histopathologic perspective, dFH-LMs often exhibit increased cellularity and nuclear atypia, features that complicate the diagnosis. Of particular concern is that these leiomyomas share important histologic features with ALMs, contributing to diagnostic uncertainty and potentially inconsistent clinical management [25,26]. This differential diagnosis is critical and extends to uterine leiomyosarcoma, where immunohistochemical panels (e.g., for p53, RB1, ATRX, PTEN) have shown utility in distinguishing malignant from benign smooth muscle tumors [27]. Given these challenges, a coherent and standardized diagnostic pathway is warranted.

The morphologic features have an empirical basis. Ubago et al. (2016) studied 60 atypical leiomyomas and defined a ‘subtype I’ enriched with FH alterations, highlighting round or oval nuclei with smooth membranes, prominent nucleoli with perinuclear halos and diffuse atypia as statistically significant features [10]. However, FH expression was not analyzed in this study. This constellation is a suitable histologic signal to suspect the presence of fumarase deficiency at the time of primary microscopic examination and may improve the selection of methods for complementary testing.

To further develop this idea and confirm existing observations by scientists, we also decided to analyze the frequency of occurrence of dFH-LM in ALM, as well as to determine the diagnostic significance of histological features associated with fumarase deficiency and the reproducibility of the main morphological criteria for the definition of dFH-LM.

Despite its exploratory nature, our study provides some insights into morphological features for the identification of dFH-LM. In our single-center cohort, more than half of the tumors initially classified as ALMs were classified as dFH-LMs by IHC (26/45; 57.7%). These results are similar to those of Miettinen et al. (2016), who found FH loss in 37.3% (68/182) of ALMs, and are consistent with the retrospective series by Kipnis et al. (2024), in which 48 of 144 atypical uterine leiomyomas were found to be FH deficient by IHC (48/144; 33.3%) [2,28].

Our study shows that eosinophilic globules, staghorn-like vessels, and the presence of diffuse nuclear atypia are statistically significant morphologic features. Although the features we describe are not specific for an FH germline mutation, we believe that the features are strongly suggestive of USMTs with FH aberration. These findings are consistent with Li et al., who later quantified the frequency of the characteristic features-staghorn-like vessels (87%), “bizarre” nuclei (81%), alveolar edema (65%), nucleoli (65%), and eosinophilic globules (56%)—supporting the diagnostic value of these morphologic features [29]. In addition, patients with dFH-LM were significantly younger than those with ALMs, which is consistent with previous clinicopathologic series [2,10,30].

Despite the increasing recognition of these morphologic markers, interobserver reproducibility in their assessment has not yet been fully evaluated. Although numerous clinicopathologic reports and reviews have emphasized eosinophilic globules as a characteristic feature of dFH-LM or ALM—often along with staghorn-like vessels, diffuse/weak fascicle architecture, and prominent eosinophilic nucleoli—to our knowledge, there are no formal metrics for interobserver agreement (e.g., Cohen’s *κ* or Fleiss’s *κ*) specifically for this feature. This gap in the literature suggests that standardized definitions and training materials would be beneficial for routine practice.

In our study, for methodological reasons, we limited ourselves to testing interobserver reproducibility for eosinophilic globules. Globules are a visually discrete, easily operationalized feature (“present/absent”, with optional semiquantification) that is repeatedly described among the characteristic morphologies of dFH-LM—typically alongside staghorn-like vessels and prominent nucleoli with perinuclear halos—and is therefore well suited for standardized matching studies [6].

A molecular study showed that nucleoli with perinuclear halos, eosinophilic globules, and staghorn-like vessels correlate with 2SC immunoreactivity, a metabolic surrogate of FH deficiency [30]. This correlation justifies the use of eosinophilic globules as a convenient anchoring feature for assessing reproducibility without the need for direct molecular testing. In contrast, several alternative candidate features (e.g., diffuse atypia, staghorn-like vessels) show recognized overlap with ALMs, reducing their specificity and potential interobserver reproducibility in blinded review. Accordingly, it makes methodological sense to favor a feature with greater visual definability for initial validation [8,31].

Therefore, it is reasonable to prioritize a feature with greater visual definability, namely eosinophilic globules, for initial validation before resorting to a broader panel in subsequent prospective studies.

Since any morphology-guided program must work reliably between observers- and not just within a single expert group- reproducibility is crucial. In our experience, interobserver agreement for eosinophilic globules reached a substantial Fleiss’s *κ* of 0.63.

There are several possible explanations for these results: firstly, these differences could be due to the binary evaluation and the anchoring context. The use of a simple presence/absence prompt (as opposed to multilevel gradations) generally increases agreement, and when raters are anchored by consistent contextual cues (e.g., concomitant features typical of FH deficiency), reliability tends to improve [32].

Secondly, in the interpretation of eosinophilic globules on H&E, both false-positive and false-negative findings are plausible and largely reflect the biological non-specificity, technical variability and context dependence of the trait. False-positive findings may arise because eosinophilic globules have been documented in a wide range of neoplastic and non-neoplastic processes (e.g., breast, pancreas, kidney), where they represent a degenerative/apoptotic or protein accumulation phenomenon rather than a lineage-specific sign. Identification of such inclusions in USMTs without appropriate context therefore risks overestimating FH-deficient morphology [9,11,12,13,33,34,35].

Thirdly, technical and interpretive artifacts-including fixation/processing issues, section thickness, fading or suboptimal H&E contrast, and digital rendering effects in telepathology—can further mimic or exaggerate globular eosinophilic material, which in turn can lead to false-positive results [36].

The study also shows that IHC can enhance diagnostic accuracy without compromising efficiency. In particular, loss of FH protein expression combined with positive 2SC staining—a surrogate for fumarate accumulation—represents a targeted IHC pairing that resolves many dubious cases prior to molecular reflex testing. In practice, morphology screening followed by FH/2SC IHC strikes a useful balance between sensitivity and specificity, referring truly equivocal or high-risk tumors for sequencing and genetic counseling [26,31].

There are several limitations to this study. First, mutational analyses were not performed, which precludes definitive molecular confirmation of FH deficiency and prevents differentiation between somatic and germline alterations. Consequently, FH/2SC IHC results were not validated against genetic testing, and the risk of false positives/negatives could not be quantified. Second, the lack of comprehensive clinical information relevant to HLRCC phenotyping—such as detailed records of cutaneous leiomyomas, systematic renal imaging results, and complete family cancer histories—for a substantial portion of our cohort is a significant limitation. This prevented robust syndromic correlation and may have introduced ascertainment bias, as we could not definitively distinguish between sporadic and germline-related cases. The absence of these data limits the generalizability of our findings to the broader HLRCC context and underscores that our study primarily addresses the histopathological differentiation of dFH-LM from ALM within uterine pathology, rather than providing a comprehensive clinicogenetic profile.

Third, although multiple leiomyomas were reported in some patients, histologic slides of all lesions were not accessible, raising the possibility that intrauterine heterogeneity went unrecognized and dFH-associated morphology was under- or overestimated. Fourth, eosinophilic globules were not validated by additional methods (e.g., special stains, ultrastructural or proteomic studies); thus, their specificity and interobserver reproducibility remain uncertain. Finally, limitation of this study is its single-center, retrospective design from a single tertiary medical center setting, which may reflect local referral patterns, pre-analytic handling, and case-mix, thereby limiting generalizability. We did not perform external validation or assess interinstitutional reproducibility; therefore, our estimates of diagnostic performance and agreement may not fully apply to laboratories that differ in fixation or processing protocols, antibody clones and staining platforms (FH/2SC), or post-analytic workflows such as scanning and display calibration. In addition, our study did not account for potential confounders such as pre-analytical variables (fixation, staining quality) or menopausal status, which might influence morphological interpretation. Future prospective studies would benefit from standardizing these technical factors and collecting comprehensive clinical data. Overall, these limitations argue for a cautious, hypothesis-generating interpretation pending confirmation in rigorously designed prospective cohorts with comprehensive genotyping, standardized IHC, complete clinical phenotyping, and systematic sampling of lesions.

Ultimately, the early and accurate categorization of dFH-LM carries significant therapeutic and genetic implications. As mechanism-based therapies targeting the vulnerabilities of FH-deficient cells continue to mature in preclinical and clinical settings, a reliable and standardized diagnostic pathway becomes paramount for identifying patients who may benefit from such targeted interventions. Beyond immediate treatment considerations, this precise diagnosis is the critical first step in identifying individuals with hereditary leiomyomatosis and renal cell carcinoma (HLRCC) syndrome, enabling proactive genetic counseling, family testing, and life-saving renal surveillance. The integration of metabolic insights with standardized morphological assessment and targeted IHC, as validated in our study, provides a robust and practical framework to achieve this diagnostic precision. Our findings, which demonstrate that reproducible morphological features are strongly correlated with the underlying molecular driver, argue compellingly for the incorporation of specific criteria—particularly for eosinophilic globules—into future diagnostic guidelines and routine practice. To build upon this foundation, future research should focus on the multi-institutional validation of these standardized morphological criteria, the development of educational tools to enhance their consistent application, and the exploration of how this integrated diagnostic approach impacts genetic referral patterns and long-term patient outcomes. Therefore, advocating for the wider application of this morphology-emphasizing, IHC-confirmed approach is not only feasible and effective but essential for advancing personalized care for patients with these distinctive tumors. [4,22,23].

**Figure 6:**
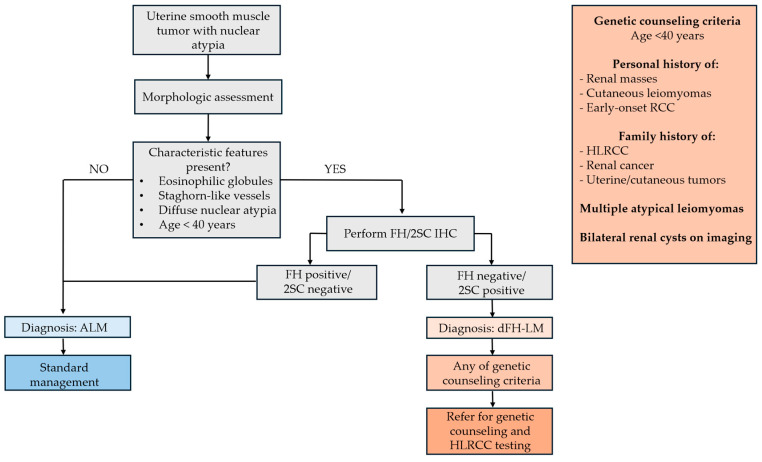
Proposed diagnostic flowchart integrating morphologic and IHC criteria.

Despite the limited cohort and the fact that these features are not pathognomonic for FH germline mutation, the findings suggest FH-aberrant USMTs. Identifying such cases is a key initial step in the diagnostic workup of young patients suspected of HLRCC. Thus, we can propose a diagnostic flowchart integrating morphologic and IHC criteria (Figure 6).

## 5. Conclusions

This study shows a high frequency of FH in tumors initially diagnosed as ALMs, confirming the complexity of the differential diagnosis of these two nosologies. It is shown that eosinophilic globules, staghorn-like vessels and diffuse nuclear atypia have a high diagnostic value and could be considered as morphologic “indicators” of dFH-LM. The substantial reproducibility of such a key feature as eosinophilic globules among pathologists makes it particularly promising for use in daily clinical practice. Based on our findings, we propose that the identification of even one of these characteristic morphological features, particularly eosinophilic globules, should trigger reflexive FH/2SC IHC testing to confirm the diagnosis and further referral of young patients for genetic counseling.

## Figures and Tables

**Table 1:** Interobserver agreement among three pathologists and concordance with FH/2SC IHC (Cohen’s kappa, 95% CI).

	Pathologist 1	Pathologist 2	Pathologist 3
Pathologist 1		0.63 (0.39–0.88)	0.70 (0.50–0.91)
Pathologist 2			0.55 (0.30–0.80)
IHC results	0.58 (0.33–0.84)	0.64 (0.40–0.88)	0.60 (0.36–0.84)

CI = Confidence interval, IHC = Immunohistochemistry.

**Table 2:** General clinical and histologic features of fumarate hydratase-deficient leiomyomas and atypical leiomyomas.

Features		dFH-LM (*n* = 26)	ALM (*n* = 19)	*p*-Value
Age (years)	Median, (Q1–Q3)	34 (27–39)	41 (38–44)	0.03 *
Tumor size (cm)	Mean, 95% CI	6.6 (5.1–8.0)	6.9 (5.1–8.7)	0.76
Recurrence		1 (3.8%)	1 (5.3%)	0.99
Multiplicity	Single	7 (26.9%)	7 (36.8%)	0.53
	Multiple	19 (73.1%)	12 (63.2%)	
Eosinophilic globules		23 (88.5%)	3 (15.8%)	0.001 *
Staghorn-like vessels		19 (73.1%)	5 (26.3%)	0.02 *
Nucleoli		15 (57.7%)	8 (42.1%)	0.67
“Bizarre” nuclei		14 (53.9%)	6 (31.6%)	0.67
“Alveolar-type” edema		16 (61.5%)	8 (42.1%)	0.67
Cellularity	Hypercellular	6 (23.1%)	9 (47.4%)	0.46
	Normocellular	20 (76.9%)	10 (52.6%)	
Distribution of nuclear atypia	Focal	4 (15.4%)	12 (63.2%)	0.01 *
	Diffuse	22 (84.6%)	7 (36.8%)	
Nuclear atypia	1+	14 (53.8%)	15 (78.9%)	0.29
	2+	11 (42.3%)	2 (10.5%)	
	3+	1 (3.8%)	2 (10.5%)	

*—*p* ≤ 0.05. *p*-values are shown are after Holm–Bonferroni adjustment (two-sided). dFH-LM = Fumarate hydratase-deficient leiomyoma, ALM = Atypical leiomyoma.

## Data Availability

Data supporting the reported results can be provided upon reasonable request.

## Abbreviations

The following abbreviations are used in this manuscript:

dFH-LM	Fumarate hydratase-deficient leiomyoma
FH	Fumarate hydratase
HLRCC	Hereditary leiomyomatosis and renal cell carcinoma
USMTs	Uterine smooth muscle tumors
MRI	Magnetic Resonance Imaging
ALM	Atypical leiomyoma
H&E	Hematoxylin and eosin
FFPE	Formalin-fixed, paraffin-embedded
IHC	Immunohistochemistry
2SC	S-(2-succino) cysteine
PPV	Positive predictive value
NPV	Negative predictive value
CI	Confidence interval

## Appendix A

**Table A1:** Eosinophilic globules in atypical leiomyoma determined by each pathologist and validated with IHC staining.

Case No.	FH	2SC	Pathologist 1	Pathologist 2	Pathologist 3
1	negative	positive	1	1	1
2	negative	positive	1	1	1
3	negative	positive	1	1	1
4	negative	positive	1	1	0
5	positive	negative	0	0	0
6	negative	positive	1	0	1
7	negative	positive	1	1	1
8	negative	positive	1	1	0
9	positive	negative	0	0	0
10	negative	positive	1	1	1
11	positive	negative	1	1	0
12	positive	negative	0	1	0
13	positive	negative	0	0	0
14	negative	positive	1	1	1
15	negative	positive	0	0	0
16	negative	positive	1	1	1
17	positive	negative	1	0	0
18	positive	negative	1	0	0
19	positive	negative	0	0	0
20	positive	negative	0	1	0
21	negative	positive	1	1	1
22	negative	positive	0	1	0
23	negative	positive	1	1	1
24	positive	negative	0	0	0
25	negative	positive	1	1	0
26	negative	positive	1	1	1
27	positive	negative	0	0	0
28	negative	positive	1	1	1
29	negative	positive	1	1	1
30	positive	negative	0	0	0
31	positive	negative	1	0	1
32	negative	positive	1	1	1
33	positive	negative	0	0	0
34	positive	negative	0	0	0
35	negative	positive	0	0	0
36	positive	negative	0	0	0
37	positive	negative	1	1	1
38	negative	positive	1	1	1
39	negative	positive	1	1	1
40	negative	positive	1	1	1
41	positive	negative	0	0	0
42	positive	negative	0	0	0
43	negative	positive	1	1	1
44	positive	negative	0	0	0
45	negative	positive	1	1	1
